# Supplementary material for: Oligomer Formation by Physiologically Relevant C-Terminal Isoforms of Amyloid β-Protein
Source: Biomolecules. 2024 Jun 28;14(7):774. doi: 10.3390/biom14070774 (PMC11274879; doi:10.3390/biom14070774)
Supplement: Supplementary file 1 [file biomolecules-14-00774-s001.zip › biomolecules-3049142-supplementary.pdf]

Supplementary Materials: Oligomer formation by physiologically-relevant C-terminal isoforms of amyloid β-protein

Rachit Pandey[1] and Brigita Urbanc[1]*

Supplementary Tables

	n=1	n=2	n=3	n=4	n=5	n=6	n=7	n=8	n=9	n=10
Aβ_{1-38}	1104	27822	20457	8235	4339	1709	2168	230	268	55
Aβ_{1-40}	197	21423	21410	11225	4631	3046	795	534	21	8
Aβ_{1-42}	56	13170	14343	10695	4417	4512	2153	1116	707	475
Aβ_{1-43}	276	11815	15220	9968	4955	3734	2386	1750	805	218

Table S1. Number of monomer and oligomer conformations derived from simulations between 20×10^6 and 40×10^6 time units, which were used in structural analyses.

	α_1	α_2	α_3	α	β	γ
Aβ_{1-38}	2.55	2.48	1.63	0.76	0.32	1.39
Aβ_{1-40}	2.62	2.62	1.56	0.81	0.28	1.37
Aβ_{1-42}	2.45	2.41	1.61	0.72	0.31	1.33
Aβ_{1-43}	2.60	2.59	1.55	0.80	0.28	1.35

Table S2. Scaling exponents α_1, α_2, and α_3 of the three principal moments of inertia, I_1, I_2, and I_3, respectively, derived from fitting the data in Fig. S6, and the resulting elongation, thickness, and volume scaling exponents: α, β, and γ.

Supplementary Figures

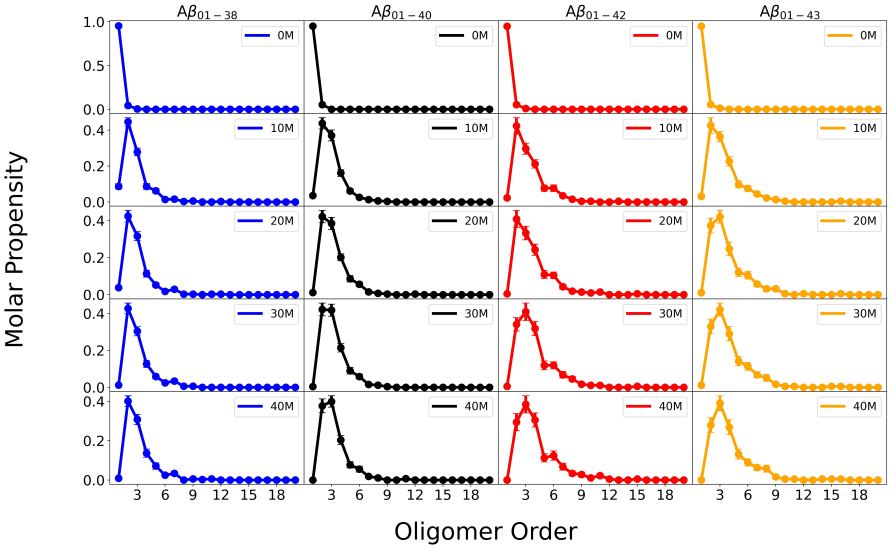

Figure S1. Time evolution of molar oligomer size distributions of Aβ_{1-38}, Aβ_{1-40}, Aβ_{1-42} and Aβ_{1-43}. Each oligomer size distribution is an average over 32 independent trajectories. The error bars correspond to SEM values.

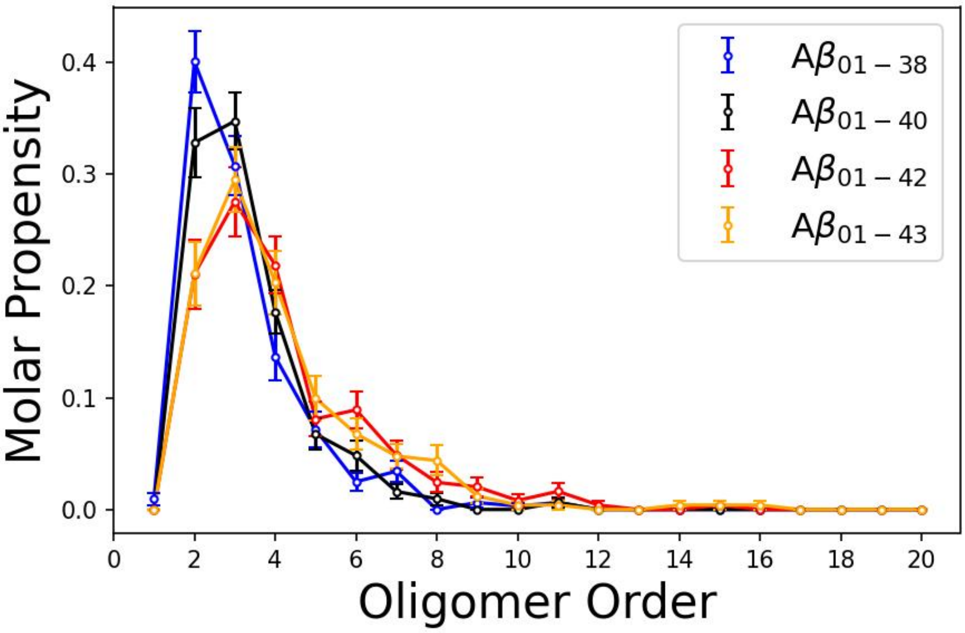

Figure S2. Steady-state molar oligomer size distributions of Aβ_{1-38}, Aβ_{1-40}, Aβ_{1-42}, and Aβ_{1-43} at 40×10^6 simulation time units. The error bars correspond to SEM values as described in *Methods*.

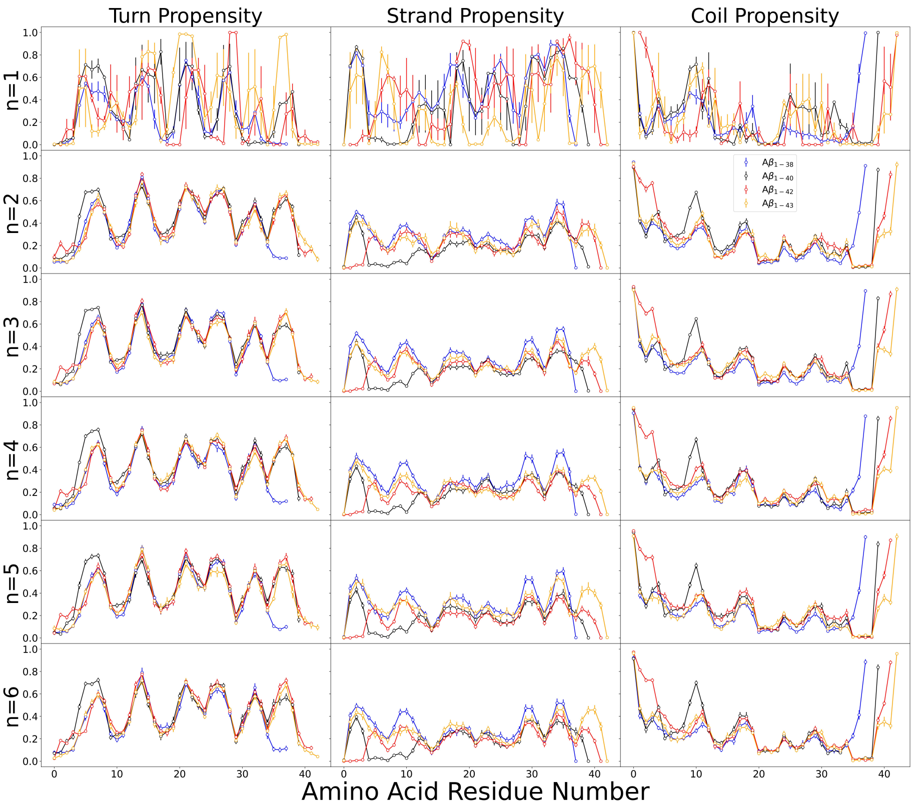

Figure S3. Average per-residue turn, strand, and coil propensities for monomers (n = 1) through hexamers (n = 6) formed by Aβ_{1-38}, Aβ_{1-40}, Aβ_{1-42}, and Aβ_{1-43} within 20×10^6-40×10^6 simulation time units. The error bars correspond to SEM values.

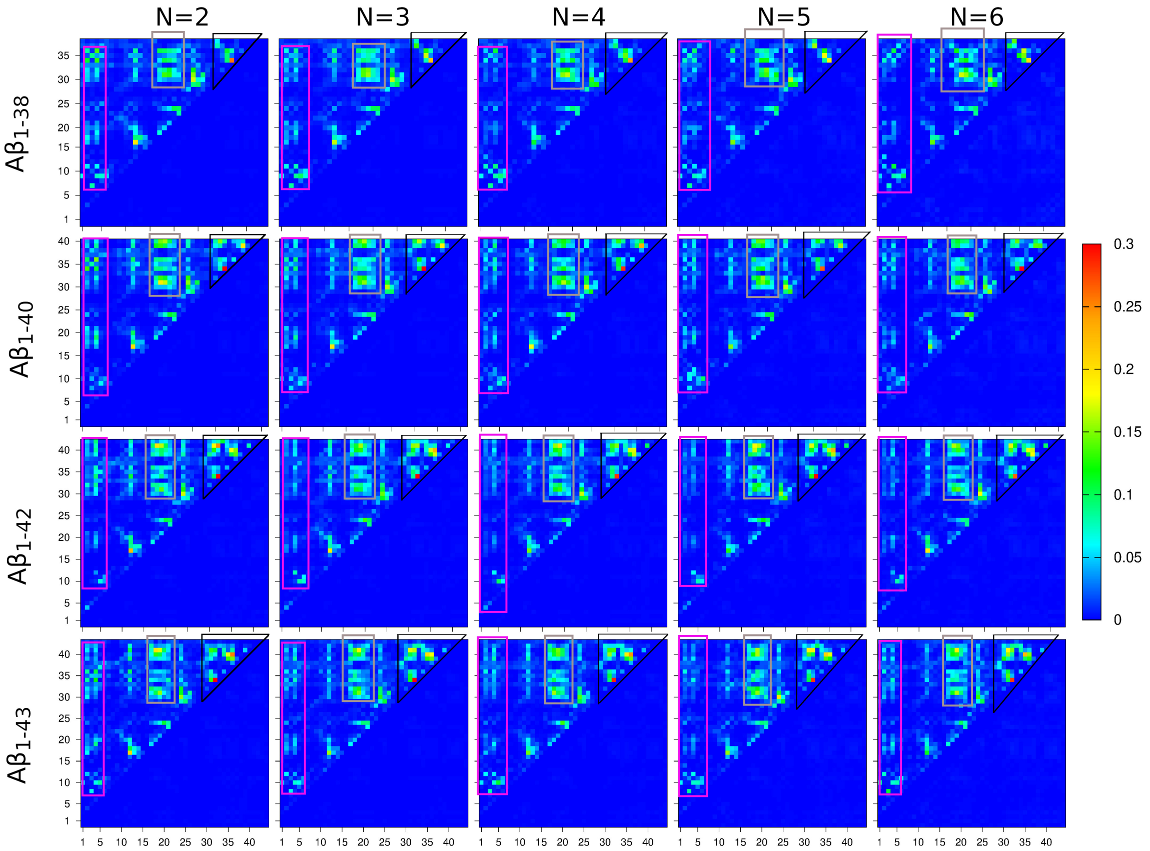

Figure S4. Intramolecular contact maps for monomers through hexamers of Aβ_{1-38}, Aβ_{1-40}, Aβ_{1-42}, and Aβ_{1-43}. Tertiary contacts of the A2-F4 region with other residues along the sequence are enclosed in a magenta rectangle. Tertiary contacts that the CHC forms with the MHR and CTR are enclosed in a gray rectangle. Tertiary contacts within the black triangle are those formed between the MHR and CTR.

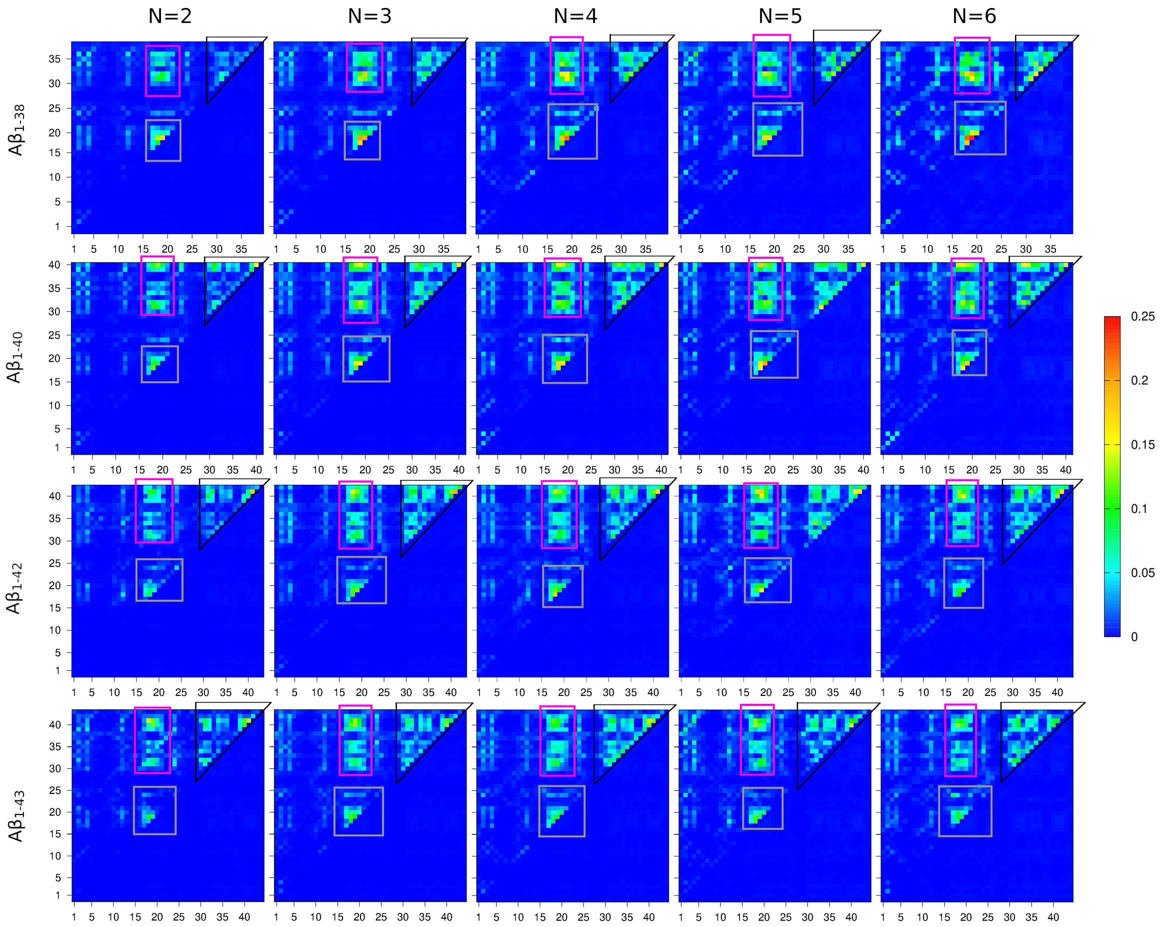

Figure S5. Intermolecular contact maps for monomers through hexamers of Aβ_{1-38}, Aβ_{1-40}, Aβ_{1-42}, and Aβ_{1-43}. Quaternary contacts between pairs of CHCs are enclosed in a gray rectangle. Quaternary contacts that the CHC forms with the MHR and CTR are enclosed in a magenta rectangle. Quaternary contacts within the black triangle are those formed between pairs of MHRs, pairs of CTRs, and between the MHR and the CTR.

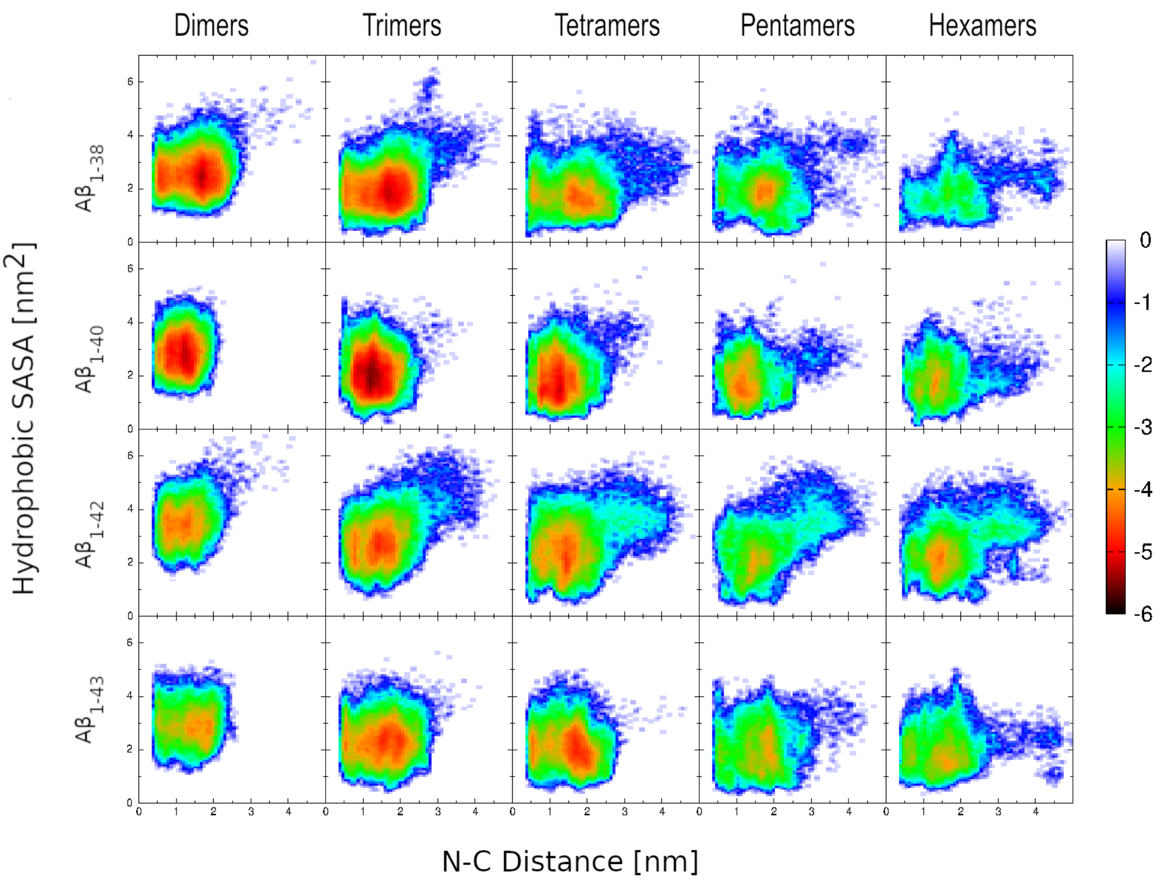

Figure S6. PMF landscapes of $A\beta_{1-38}$, $A\beta_{1-40}$, $A\beta_{1-42}$, and $A\beta_{1-43}$ monomers through hexamers. Hydrophobic SASA and N-C distance were used as the two reaction coordinates. The y-axis shows the hydrophobic solvent accessible surface area in nm^2, the x-axis shows the N-terminal to C-terminal distance in nm, and the color scale shows the PMF and is in units of k_BT.

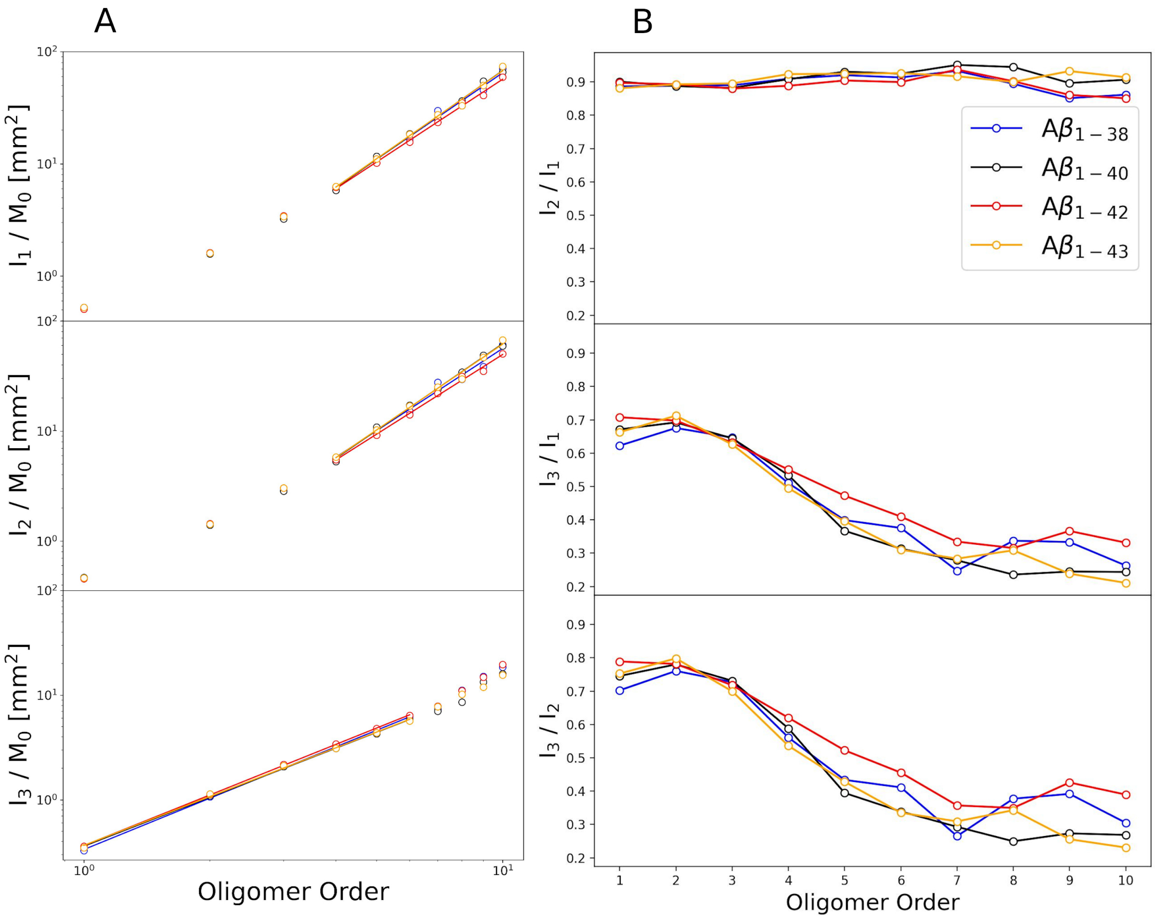

Figure S7. (A) The principle moments of inertia I_1, I_2, and I_3 versus the size of Aβ_{1-38}, Aβ_{1-40}, Aβ_{1-42}, and Aβ_{1-43} oligomers, normalized by the mass of a monomer, M_0. (B) The ratios of the principal moments of inertia: I_2/I_1, I_3/I_1 and I_3/I_2 versus the oligomer size for each of the four Aβ variants.
